# Retinal vasculitis in two pediatric patients with systemic lupus erythematosus: a case report

**DOI:** 10.1186/1546-0096-11-25

**Published:** 2013-06-04

**Authors:** Katherine J Donnithorne, Russell W Read, Robert Lowe, Peter Weiser, Randy Q Cron, Timothy Beukelman

**Affiliations:** 1 Department of Ophthalmology, University of Alabama at Birmingham, Birmingham, AL, USA; 2 Department of Pediatrics, Division of Rheumatology, University of Alabama at Birmingham, Birmingham, AL, USA

**Keywords:** Systemic lupus erythematosus (SLE), Retinal vasculitis, Branch retinal artery occlusion

## Abstract

We report two pediatric female patients with systemic lupus erythematosus (SLE) who presented with decreased vision. Both patients were found to have retinal vasculitis and occlusive disease. The first patient also presented with vitreous hemorrhage and later non-arteritic ischemic optic neuropathy. She was treated with panretinal photocoagulation and steroid therapy and later in her disease course was treated with rituximab and cyclophosphamide. Her vision remained decreased. The second patient was treated with rituximab and monthly cyclophosphamide infusions early in her disease course, and her vision improved dramatically. The difference in the presentations and outcomes of these two pediatric patients with SLE highlights the spectrum of severity of SLE retinopathy. We suggest that early recognition of disease and early intervention with B-cell depletion therapy in addition to a traditional cytotoxic agent should be considered in pediatric patients with SLE and occlusive retinopathy.

## Background

Systemic lupus erythematosus (SLE) is a relatively rare auto-immune disorder that may affect multiple organ systems, including the eye. The most common ophthalmic manifestation of SLE is keratoconjunctivitis sicca (dry eye syndrome). The most common intraocular manifestation is retinopathy with microangiopathy due to arterial occlusion [1,2], but other ophthalmic manifestations may occur as well (e.g., uveitis, scleritis, episcleritis, choroidopathy). The incidence of SLE-associated retinopathy has been reported to be 7-29% among adult patients [2,3], while occlusive retinopathy has been found in approximately 3-11% of SLE patients [3,4]. Retinopathy appears to be less common among children with SLE, although to our knowledge, no incidence studies have been published.

Retinal vasculitis can involve both the arteries and the veins; however, the arteries are more frequently affected in SLE [5]. Ophthalmic examination findings of retinal vasculitis include the presence of vitreous cells and sheathing of the retinal blood vessels. Occlusive retinal vasculitis may manifest with cotton wool spots (representing nerve fiber layer infarcts), intraretinal hemorrhages, optic nerve head swelling, and macular edema [5-7]. Other findings, which may present later in the course of the disease, include microaneurysms, neovascularization related to ischemia, and tractional retinal detachment. Fluorescein angiography–in which sodium fluorescein dye is injected intravenously and retinal photographs are taken as the fluorescein circulates through the retinal vessels– highlights the leakage produced by damage to vessel walls (which form the inner blood-retinal barrier) [5]. Optical coherence tomography is a noninvasive diagnostic imaging modality used for characterizing macular edema.

SLE retinopathy has been correlated with active lupus, antiphospholipid antibody syndrome (APS), and central nervous system lupus [7]; thus, its recognition is important for assessment of morbidity and prognosis. There are few published reports of SLE-associated retinopathy in the pediatric population. In this report we present and discuss two pediatric patients with SLE who developed retinal vasculitis.

## Case presentations

### Patient #1

A 16-year-old African American female was diagnosed with SLE in August 2010, after initially presenting to the pediatric rheumatology clinic with abdominal pain, nausea, vomiting, headache, fever, weight loss, and rash. She was found to be ANA positive, with positive antibodies to SS-A, SS-B, RNP, and dsDNA. Anti-cardiolipin antibody, anti-β2 glycoprotein 1 antibody, and lupus anticoagulant studies were negative. At that time she was treated with hydroxychloroquine and a tapering dose of oral prednisone.

**Figure 1:**
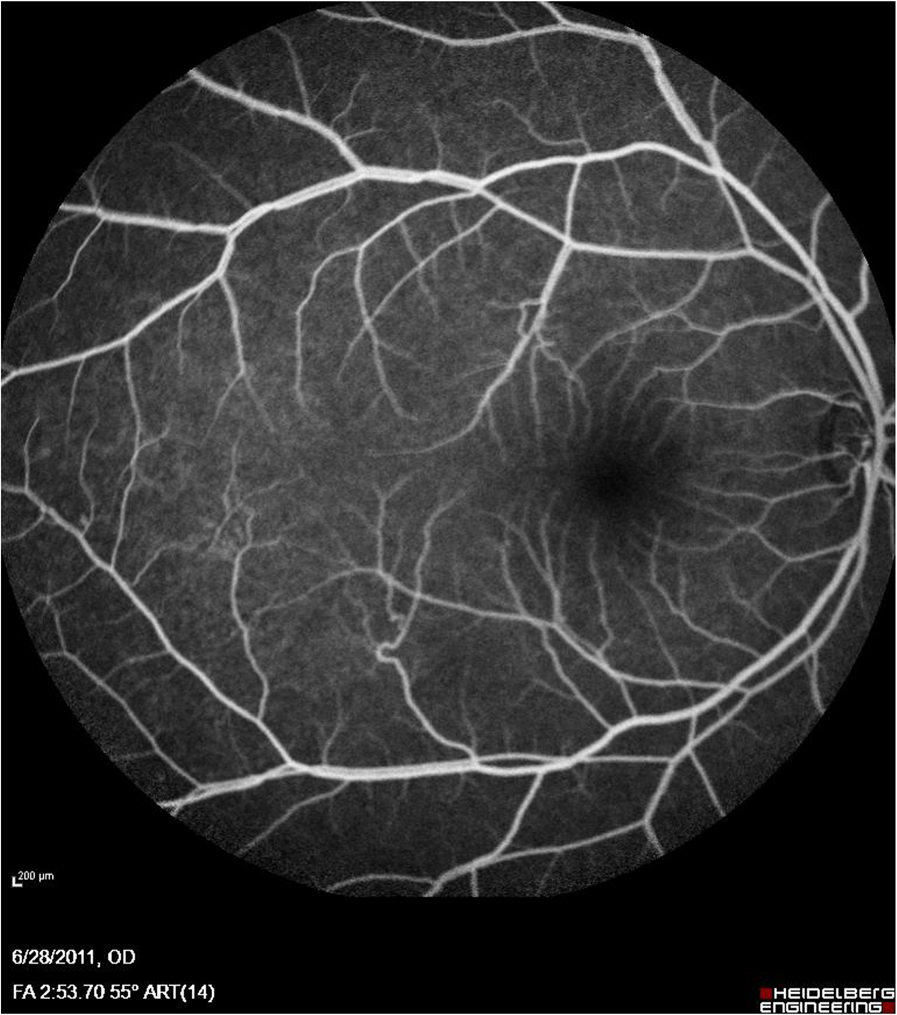
Fluorescein angiography of Patient #1 showing normal fundus of right eye.

**Figure 2:**
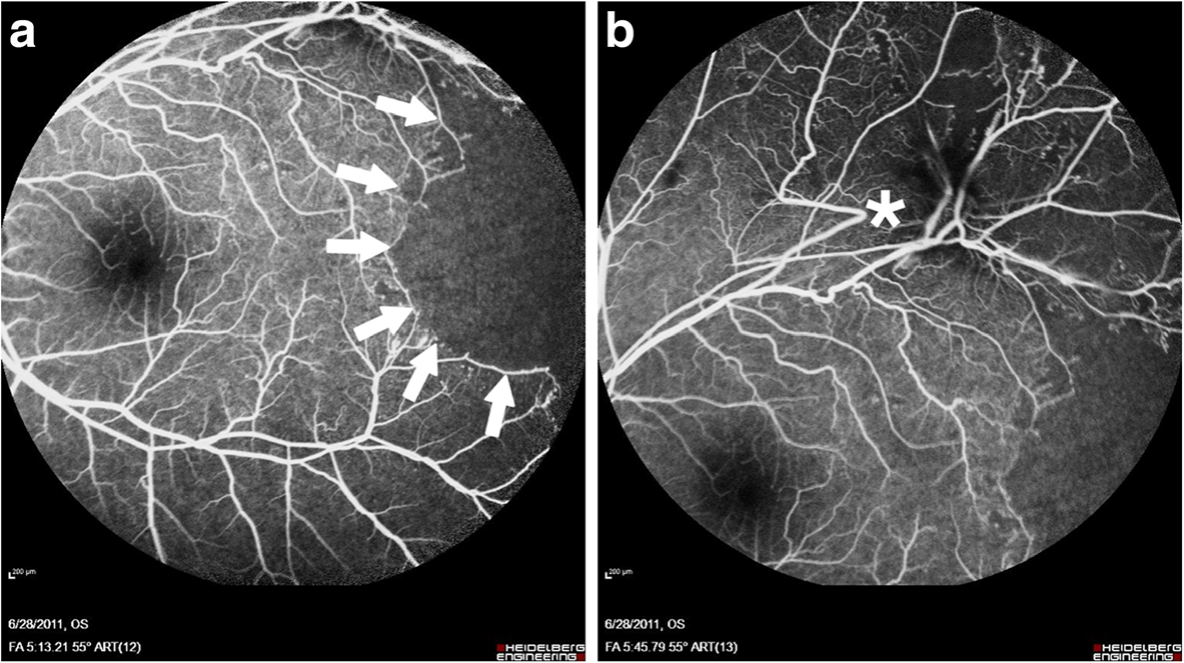
Fluorescein angiography of Left eye of Patient #1 showing [a] large area of capillary non-perfusion (outlined by solid arrows) and [b] superotemporal view showing distortion of vessels (asterisk) indicating traction, with area of non-perfusion.

She had no vision complaints until June 2011, when she developed acute vision loss in the left eye. Her vision in the right eye was 20/20 and count fingers at 2 feet in the left eye. She was referred to a retina specialist, who determined that she had a branch retinal artery occlusion with macular ischemia, retinal neovascularization, vitreous hemorrhage, and tractional retinal detachment in the left eye. The patient also had evidence of non-arteritic ischemic optic neuropathy (NAION). Later that month, the patient’s vision was improved to 20/400 in the left eye, and she underwent fluorescein angiography to identify areas of retinal ischemia (see Figures 1 and 2). She also received panretinal photocoagulation in the left eye.

In August 2011, the patient complained of a “black spot” in the superior medial region of her left eye. She began mycophenolate mofetil (MMF) for lupus retinopathy, which was increased to 1,000 mg twice daily, together with oral prednisone 20 mg daily. She underwent a contrast-enhanced MRI of the brain, which showed no evidence of vasculitis or other pathologic process.

By October 2011, she maintained stable vision of 20/20 in the right eye and 20/400 in the left eye. Vitreous hemorrhage in the left eye persisted and her optic nerve now showed 1+ pallor. The retinal neovascularization had regressed. Later that month, her MMF dose was increased to 1,500 mg twice daily.

In April 2012, the patient returned to the retina specialist with an improvement in vision to 20/200 + 1 in the left eye. Her dilated fundus examination remained unchanged. She was found to have an afferent papillary defect in the left eye as a result of the non-arteritic optic neuropathy.

In June 2012, with the hope of gaining better control of her SLE and preventing further vision loss, the patient was treated with two rituximab infusions (750 mg/m^2^) given 2 weeks apart and initiated monthly cyclophosphamide infusions (750 mg/m^2^ per dose) with concurrent pulse methylprednisolone infusions (1,000 mg). At that time MMF was discontinued while hydroxychloroquine and prednisone were continued. She achieved successful B-cell depletion following the rituximab infusions. In November 2012 she returned to ophthalmology. Her vision remained 20/20 in the right eye and 20/200-1 with pinhole in the left eye. On exam she continued to have an afferent pupillary defect in the left eye and a pale optic nerve. The patient had evidence of traction in the retina temporally with neovascularization and vitreous hemorrhage. She again underwent panretinal photocoagulation to fill in areas in the far retinal periphery with the aim to halt further ischemia-induced neovascularization.

### Patient #2

A 13-year-old African American female patient presented to the emergency department in September 2011 with fever, malar rash, and arthralgias. She was found to have elevated lipase (491 Units/L; normal 10–180), amylase (191 Units/L; normal 30–100), ESR (75 mm/hour; normal 0–20), CRP (1.15 mg/dL; normal <1.0), and CPK (344 Units/L; normal 30–170), Babesia titers, and Ehrlichiosis titers. Rheumatology was consulted, and the patient was found to be ANA positive, with antibodies to Smith, dsDNA, RNP, and SS-A. Testing for anti-cardiolipin antibody, anti-β2 glycoprotein 1 antibody, and lupus anticoagulant was negative. Repeat Ehrlichiosis titers were negative and her fever had resolved without specific antimicrobial therapy. Repeat Babesia titers again revealed elevated IgG and IgM levels. The patient was diagnosed with SLE. Immunosuppression therapy was initially held until she completed a course of azithromycin and atovaquone for possible Babesiosis.

**Figure 3:**
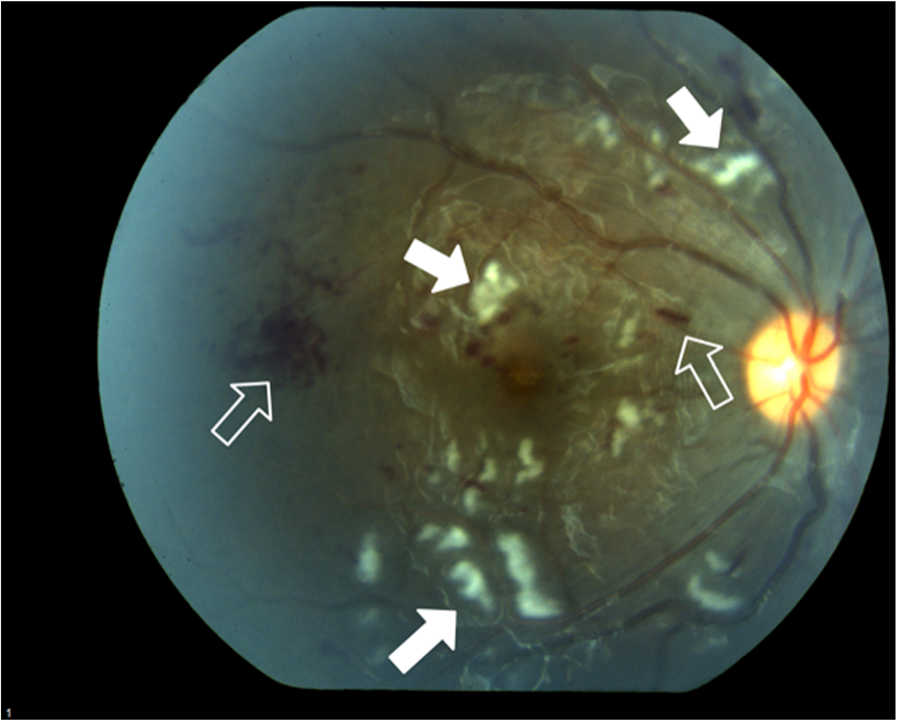
Photograph of right fundus of Patient #2 showing scattered nerve fiber layer infarcts (solid arrows) and retinal hemorrhage (outlined arrows).

Later that month, the patient complained of vision changes in her right eye and was evaluated in the ophthalmology clinic, where her vision with correction was 20/80 in the right eye and 20/20 in the left eye. On dilated fundus examination a glistening sheen was noted over the macula in the right eye, with intraretinal hemorrhages and cotton wool spots, without neovascularization. It was concluded that she had retinal vasculitis associated with SLE in the right eye. She was referred to a retinal specialist, who noted multiple cotton wool spots in the right eye associated with two prominent branch retinal artery occlusions along the superotemporal arcade. A single cotton wool spot was visualized in the left posterior pole (Figure 3). The patient was found to have an elevated ESR (79 mm/hour) and was hospitalized for further management. The patient underwent MRI /MRA brain, which showed no evidence of vasculitis. During hospitalization, the patient received 3 doses of pulse methylprednisolone (1,000 mg) as well as the first doses of infusions of rituximab (750 mg/m^2^, 2 infusions given 2 weeks apart) and monthly cyclophosphamide (750 mg/m^2^). She was also treated with a slow tapering dose of oral prednisone.

**Figure 4:**
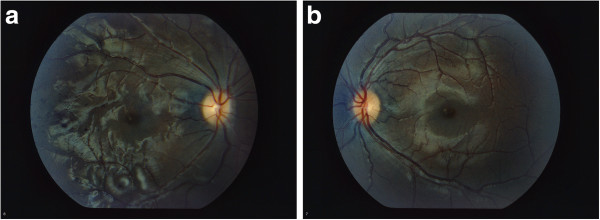
Photographs of fundi of Patient #2 showing [a] Right fundus with improvement in changes caused by vasculitis and [b] normal appearing fundus on the Left.

On ophthalmologic follow-up in November 2011, the patient’s vasculitic changes in her right macula were judged to be 80% resolved (Figure 4). Her vision had improved to 20/40 in the right eye, and her left eye vision had remained stable at 20/20. She completed 7 total monthly infusions of cyclophosphamide, followed by initiation of MMF 500 mg twice daily (300 mg/m^2^/dose; dose not further increased due to lymphopenia) and continued hydroxychloroquine 200 mg daily.

In April 2012, the patient returned to the retinal specialist. Her right eye vasculitis had almost completely resolved, with only subtle vascular changes visualized. Her vision was 20/30 in the right eye and 20/20 in the left eye with immunosuppressive therapy, not requiring laser photocoagulation therapy or other ophthalmologic interventions. The patient remains stable and continues to be treated with hydroxychloroquine and MMF.

## Conclusions

Among adults, retinopathy is reported to be a relatively common manifestation of SLE. Retinal vasculitis may precede the diagnosis of SLE, with visual loss being the presenting symptom [8]. The findings often related to SLE-associated retinopathy reflect vascular damage, manifested by cotton wool spots, vascular hemorrhages, and vasculitis. Some theories suggest that the underlying disease process of retinopathy of SLE involves microvascular occlusion associated with circulating immune complexes [9].

Retinal vasculitis is caused by an inflammatory process and manifests as perivascular exudates and leakage of dye along the veins, arteries, and capillaries on fluorescein angiography [3,10,11]. SLE retinal vasculitis often manifests as an arteritis associated with microinfarction, which then results in leakage of dye surrounding the vessel walls [8].

A less common ophthalmic manifestation of SLE is occlusive vascular disease, including retinal artery, arteriole, or vein occlusion. The pathological process likely involves immune complex deposition in retinal vessels, leading to thrombosis [12]. Occlusion of the central retinal artery manifests as sudden painless vision loss. Without rapid restoration of circulation, the vision loss is likely permanent due to infarction of the inner retina. In a branch retinal artery occlusion, a permanent visual field defect that corresponds to the infarcted vessel often remains. Both occlusions often result in edema and ischemia-driven neovascularization. Neovascularization is accompanied by contractile tissue which may result in a tractional retinal detachment. Separation of the vitreous gel may tear the fragile new vessels, resulting in vitreous hemorrhage [9,13].

Furthermore, studies have demonstrated the correlation between the presence of antiphospholipid antibodies and retinopathy [8,13,14]. Anti-phospholipid antibody syndrome (APS) is associated with arterial and venous thrombosis, and Asherson, *et al*. described that patients with SLE with elevated antiphospholipid antibody levels have an increased risk of developing vaso-occlusive ocular disease [13]. Tests of anti-phospholipid antibodies should likely be performed in all patients with SLE as well as in patients that present with vaso-occlusive retinopathy.

As the ocular manifestations of SLE are often associated with active systemic disease manifesting in other organ systems, treating the systemic disease may result in improvement of ocular disease. The mainstay of treatment for SLE retinopathy is similar to the treatment of SLE in general and involves the use of systemic glucocorticoids plus other immunosuppressant therapies [15,16]. In the acute SLE-associated retinopathy setting, patients are often treated with intravenous steroid pulse therapy plus a cytotoxic agent, such as cyclophosphamide. Once disease control is attained, low-dose systemic glucocorticoids and hydroxychloroquine are often continued long-term to potentially prevent flares of disease.

Local targeted therapies for SLE retinopathy may also be of benefit. Pan-retinal photocoagulation (PRP) places thermal burns via laser in the peripheral retina to decrease the metabolic demand of retinal tissue, inducing a regression of neovascularization and is often employed to control perivascular leakage associated with lupus retinopathy. In addition, intravitreal anti-vascular endothelial growth factor injections can be highly effective in reducing macular edema and retinal neovascularization [17].

Multiple uncontrolled studies have demonstrated the benefit and relative safety of rituximab, a chimeric monoclonal antibody that depletes CD20+ B-cells, for the treatment of severe or refractory SLE [18-21]. Unfortunately, randomized controlled trials have not substantiated this observed effectiveness of rituximab [22,23]. Uncontrolled studies have demonstrated the effectiveness of rituximab specifically for the treatment of retinal vasculitis, particularly in patients with Behcet’s disease [24,25]. A recent case report described the rapid improvement in bilateral retinal vasculitis in a woman with SLE following rituximab therapy [26].

The two pediatric patients described here had different outcomes. In the second patient described, immunosuppression with pulse methylprednisolone in addition to rituximab and cyclophosphamide infusions were administered early in the disease course, while the first patient received rituximab and cyclophosphamide approximately one year after initial visual symptoms. The second patient’s vision dramatically resolved, while the first patient’s vision improved moderately from initial presentation but remained limited. While both patients showed evidence of retinal vasculitis and branch retinal artery occlusions despite being negative for antiphospholipid antibody syndrome, only the first patient developed retinal neovascularization, warranting PRP. In addition, this patient’s clinical course was complicated by ischemic optic neuropathy.

The difference in the ophthalmic manifestations, disease progression, and outcomes of these two pediatric patients with SLE highlights the spectrum of severity of SLE retinopathy. Early recognition of retinal changes as well as early rheumatologic and ophthalmologic intervention may result in better outcomes. It is possible that patient #2 had a superior visual outcome due to earlier treatment with cyclophosphamide and rituximab. We suggest that early intervention with B-cell depletion therapy in addition to a traditional cytotoxic agent should be considered in pediatric patients with SLE and occlusive retinopathy.

This study reports two cases of pediatric patients with SLE who presented with retinal vasculitis and occlusive disease. Future studies are warranted to improve therapeutic measures for patients with SLE who present with ocular disease.

## Consent

Written informed consent was obtained from the patients for publication of this Case Report and any accompanying images. A copy of the written consent is available for review by the Editor-in-Chief of this journal.

## Abbreviations

SLE: Systemic lupus erythematosus; APS: Antiphospholipid antibody syndrome; ESR: Erythrocyte sedimentation rate; NAION: Non-arteritic ischemic optic neuropathy; ANA: Antinuclear antibody; MMF: Mycophenolate mofetil; PRP: Panretinal photocoagulation.

## Competing interests

The authors declare that they have no competing interests.

## Authors’ contributions

KJD collected and reviewed patient data and drafted the manuscript. RWR contributed to the interpretation of patient data and helped draft the manuscript. RL participated in data acquisition and care of the patients. PW participated in data acquisition and care of the patients. RQC contributed to the conception and design of the study and helped draft the manuscript. TB conceived of the study and designed and coordinated the study and helped draft the manuscript. All authors critically revised the manuscript and read and approved the final manuscript.
